# Long Non-coding RNA: Insight Into Mechanisms of Alzheimer's Disease

**DOI:** 10.3389/fnmol.2021.821002

**Published:** 2022-01-14

**Authors:** Zhen Lan, Yanting Chen, Jiali Jin, Yun Xu, Xiaolei Zhu

**Affiliations:** ^1^Department of Neurology, Nanjing Drum Tower Hospital, Clinical College of Nanjing Medical University, Nanjing, China; ^2^The State Key Laboratory of Pharmaceutical Biotechnology, Department of Neurology, the Affiliated Hospital of Nanjing University Medical School, Nanjing Drum Tower Hospital, Nanjing University, Nanjing, China; ^3^Institute of Brain Sciences, Nanjing University, Nanjing, China; ^4^Jiangsu Key Laboratory for Molecular Medicine, Medical School of Nanjing University, Nanjing, China; ^5^Nanjing Neuropsychiatry Clinic Medical Center, Nanjing, China

**Keywords:** long non-coding RNA, Alzheimer's disease, amyloid beta, tau phosphorylation, mitochondrial dysfunction, oxidative stress, synaptic dynamics, biomarker

## Abstract

Alzheimer's disease (AD), a heterogeneous neurodegenerative disorder, is the most common cause of dementia accounting for an estimated 60–80% of cases. The pathogenesis of AD remains unclear, and no curative treatment is available so far. Increasing evidence has revealed a vital role of non-coding RNAs (ncRNAs), especially long non-coding RNAs (lncRNAs), in AD. LncRNAs contribute to the pathogenesis of AD via modulating amyloid production, Tau hyperphosphorylation, mitochondrial dysfunction, oxidative stress, synaptic impairment and neuroinflammation. This review describes the biological functions and mechanisms of lncRNAs in AD, indicating that lncRNAs may provide potential therapeutic targets for the diagnosis and treatment of AD.

## Introduction

Alzheimer's disease (AD), a main cause of dementia and one of the most costly and lethal diseases (2021), is clinically characterized by progressive memory deterioration or other cognitive dysfunction, which ultimately needs full-time medical care. A cross-sectional study has shown that the overall prevalence of dementia achieves 6.0% in 2020 (3.9% for AD), representing 15.07 million individuals aged over 60 years suffered dementia in China (Jia et al., 2020). Moreover, dementia has become the second largest cause of death in individuals aged more than 70 years after ischemic heart disease (Collaborators, 2019). AD is generally divided into two groups, namely the late onset of AD (LOAD) and the early onset of AD (EOAD). EOAD, also called familial AD, is closely correlated to mutations in amyloid precursor protein (APP) and the presenilin1/2 genes. The mutations lead to the dysfunction of APP processing and induce the excessive production of amyloid-beta (Aβ). However, these genes account only for near 11% of EOAD and 0.6% of all cases of AD (Karch and Goate, 2015). LOAD, also called sporadic AD, is the majority of AD cases. The most well-known genes correlating with LOAD are apolipoprotein genotype E4 (APOE4) and triggering receptor expressed on myeloid cells 2 gene (TREM2) (Ulland and Colonna, 2018; Zhao et al., 2018).

**Figure 1:**
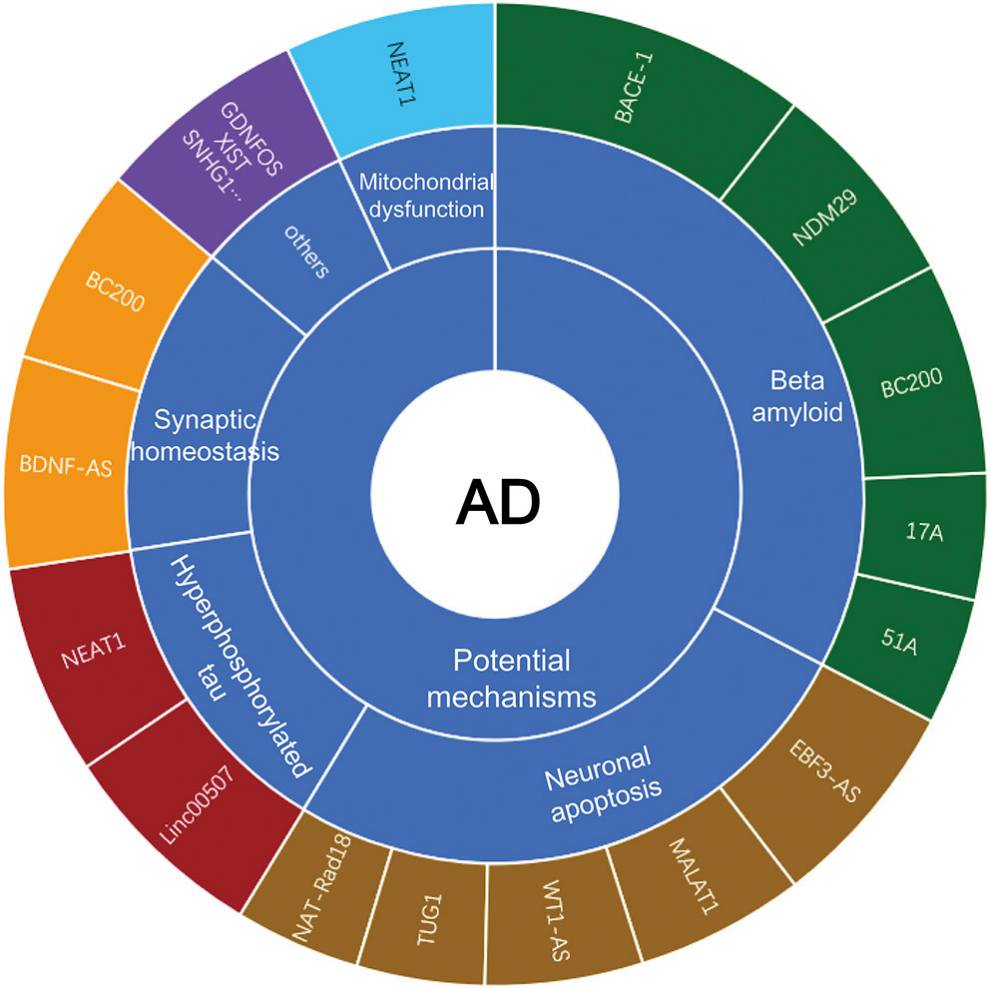
LncRNAs in the mechanisms of AD.

With the recent advancement of transcriptome-wide profiling approach, numerous of non-coding RNAs (ncRNAs) have been identified. The long non-coding RNAs (lncRNAs), which are long transcripts (>200 nucleotides in length) without apparent protein-coding capacity, have received increasing attention and are expected to be novel epigenetic regulators of gene expression at transcriptional and post-transcriptional levels (Mercer et al., 2009; Briggs et al., 2015; Zhang et al., 2019b; Karakas and Ozpolat, 2021). LncRNAs modulate chromatin functions by interaction with DNA, RNA and protein, and regulate the transcription of target genes *in cis* or *in trans* in the nucleus. In addition, lncRNAs function as miRNA sponges to suppress the miRNA availability to mRNAs in the cytosol (Statello et al., 2021). LncRNAs are widely expressed in brains and affect the proliferation, survival, metabolism and differentiation of neuronal cells, which is considered to contribute to the pathogenesis of AD (Wu et al., 2013). Mounting evidence has shown that lncRNAs are aberrantly expressed in AD progression, and modulate Aβ plague formation, tau hyperphosphorylation, neuroinflammation and neuronal apoptosis (Luo and Chen, 2016; Zhou et al., 2021). However, the underlying mechanisms of lncRNAs in AD have not yet been elucidated. Herein, we will summarize the well-characterized lncRNAs in AD (Figure 1), highlighting their potential roles in the disease pathogenesis.

## LncRNA and Aβ Accumulation

### Aβ and AD

Although the causality between Aβ and AD remains controversial, it is generally considered that Aβ may be the trigger of AD pathogenesis. In the amyloidogenic pathway, Aβ is produced through sequential cleavage of APP by β-secretase (β-site APP cleaving enzyme 1, BACE-1) and γ-secretase to produce Aβ_1−42_. In non-amyloidogenic pathway, APP is cleaved by α-secretase and γ-secretase to produce secreted amyloid precursor protein α (sAPPα), p3 and APP intracellular domain (AICD) (Morris et al., 2014; Soria Lopez et al., 2019). Aβ oligomers may trigger secondary or downstream events, such as the hyperphosphorylation of tau, synapse dysfunction and loss, inflammation, oxidative stress, and excitotoxicity, while Aβ plaques alone are not responsible for memory impairments observed in AD (Thal and Fandrich, 2015; Scheltens et al., 2016). Interestingly, recent research shows Aβ may work as an anti-microbial peptide and therefore potentially acts to combat infiltrating infectious agents (Moir et al., 2018). On June 7, 2021, aducanumab, a monoclonal antibody targeting amyloid protein, is approved to treat AD by the US Food and Drug Administration (FDA), which has sparked global debate, and further clinical trials are needed in the future (Alexander et al., 2021; Kuller and Lopez, 2021; Mullard, 2021).

### Beta-Site Amyloid Precursor Protein Cleaving Enzyme 1 Antisense Transcript Promotes Aβ Production

BACE1-AS is a conserved 2 KB non-coding antisense transcript that is transcribed from the antisense strand of the BACE1 gene locus on chromosome 11 (11q23. 3), and includes 104 nucleotides of full complementarity to human BACE1 mRNA (Faghihi et al., 2008; Kandalepas and Vassar, 2014). BACE1-AS promotes BACE1 expression at both mRNA and protein levels, which enhances APP cleavage and alters the pattern of Aβ aggregation (Li et al., 2019; Zeng et al., 2019). BACE1-AS is upregulated in peripheral blood samples and brain regions including cerebellum, hippocampus and entorhinal cortex in AD patients (Faghihi et al., 2008; Fotuhi et al., 2019). Interestingly, the accumulation of Aβ_1−42_ further increases BACE1-AS expression, driving APP processing cascade in a feed-forward manner (Faghihi et al., 2008; Li et al., 2019). The neuronal RNA-binding protein HuD interacts with BACE1-AS and increases its level, and subsequently promotes BACE1 expression and Aβ production (Kang et al., 2014). Cellular stimuli, including serum starvation, Aβ_42_ and H_2_O_2_ treatment, induce the upregulation of BACE1-AS under high glucose concentration (Boland et al., 2008; Faghihi et al., 2008; Liu et al., 2014). Knockdown of BACE1-AS by siRNA promotes the survival of primary neurons, and improves learning and memory functions of AD mice through inhibiting the expression of BACE1, APP and p-tau (Zhang et al., 2018b; Li et al., 2019).

### 51A Enhances Aβ Formation

LncRNA 51A maps in antisense configuration to the sortilin-related receptor 1 (SORL1) gene, which induces a splicing shift of SORL1 from the synthesis of SORL1 variant A to an alternatively spliced protein form. SORL1 participates in the trafficking of APP through endocytic and secretory compartments (Willnow et al., 2010; Barthelson et al., 2020), and decreased SORL1 shifts APP from the retromer-recycling endosome pathway to the β-secretase cleavage pathway, leading to increased production and accumulation of Aβ (Sager et al., 2007; Verheijen et al., 2016). Recent studies reveal that 51A is increased in the plasma and brains of AD patients compared that in controls, and indicate a negative correlation with the Mini-Mental State Examination (MMSE) scores (Luo and Chen, 2016; Garofalo et al., 2021).

### 17A Increases the Ratio of Aβ_x-42_ vs. Aβ_x-40_

LncRNA 17A is a 159 nucleotides lncRNA synthesized by RNA polymerase III, and localizes to intron 3 of the human G-protein-coupled receptor 51 gene (GPR51, GABA B2 receptor). The synthesis of 17A leads to the maturation of GABAB R2 mRNA, which induces alternative GPR51 splicing and eventually impairs GABA B-mediated signaling. The level of 17A is increased in the cerebral tissues derived from AD patients with an increased ratio of Aβ_x−42_ vs. Aβ_x−40_ (Massone et al., 2011). Overexpression of 17A in cultured neuronal cells amplifies the Aβ_42_ to Aβ_40_ ratio and promotes apoptosis (Wang et al., 2019b). All these data indicate that 17A overexpression may lead to an altered Aβ secretion and play a vital role in AD progression.

### Brain Cytoplasmic 200 Promotes Aβ Accumulation

BC200 is a polyadenylated 200 nucleotides primate neuron-specific ncRNA that is transcribed by RNA polymerase III. BC200 acts as a local translational modulator by inhibiting translation in postsynaptic dendritic microdomains, which eventually maintains the plasticity of neuron. BC200 is upregulated in specific brain areas and is increased with disease progression in AD, while it shows a steady decline in normal aging (Sosińska et al., 2015). Moreover, the overexpression of BC200 in AD is accompanied with distribution changes, including dendritic mislocalization of the transcript and accumulation of BC200 in the perikaryon (Sosińska et al., 2015; Shin et al., 2017), which has been proposed to be a starting point for the neurodegenerative changes, and eventually leads to Aβ production and amyloid deposition. In addition, BC1, a potential analog of BC200 in mice, induces APP mRNA translation through fragile X syndrome protein (FMRP), and the dysfunction of BC1 or BC1-FMRP association in AD mice impedes the aggregation of Aβ in the brain and protects against spatial learning and memory deficits (Mus et al., 2007).

### Neuroblastoma Differentiation Marker 29 Promotes Aβ Secretion

NDM29 is a lncRNA transcribed by RNA Pol III, and promotes neuroblastoma cell differentiation to a non-malignant neuron-like phenotype (Castelnuovo et al., 2010; Zhang et al., 2018a). NDM29 is upregulated in postmortem cerebral cortex from AD patients (Massone et al., 2012). NDM29 overexpression promotes the amyloidogenic processing of APP and leads to the increase of Aβ secretion and Aβ_x−42_/Aβ_x−40_ ratio (Massone et al., 2012).

## LncRNA and Tau Hyperphosphorylation

### Tau Hyperphosphorylation and AD

Tau protein is encoded by the microtubule-associated protein tau (MAPT) gene that is located on chromosome 17 in human and chromosome 11 in mice (Andreadis, 2006; Barbier et al., 2019), and plays a pivotal role in binding and stabilizing microtubules by promoting tubulin assembly to regulate the function of neurons. The abnormal hyperphosphorylation of tau alters its charge and conformation and exposes the microtubule-binding domain, leading to self-oligomerization of tau protein and forming the paired helical filaments (PHF). The aggregation of tau and PHF eventually results in the formation of neurofibrillary tangles (NFTs) (Iqbal et al., 2016; Duan et al., 2017; Guo et al., 2017). Beyond hyperphosphorylation, tau protein is also post-translationally modified through truncation, glycosylation, glycation, ubiquitination, nitration, methylation, lipoperoxidation, sumoylation, and acetylation, all of which are involved in the etiology of AD and other tauopathies (Iqbal et al., 2016). On the other hand, tau phosphorylation is regulated by a balance between phosphatase activity and tau kinase (Massone et al., 2012; Martin et al., 2013a). The number of NFTs rather than Aβ are correlated with the severity of cognitive impairment in AD patients (Giannakopoulos et al., 2003). Moreover, the distribution and accumulation of tau within synapse impairs synaptic transport and signaling pathways, leading to dysfunction and even loss of synapses in AD patients (Pooler et al., 2014; Dejanovic et al., 2018; John and Reddy, 2021). Similarly, tau oligomers are toxic to synapses and can cause synaptic impairment prior to the NFTs (Dejanovic et al., 2018). Notably, there is an intense crosstalk between Aβ and tau. Aβ exerts its toxicity at least in part through tau and the Aβ-dependent pathologies can be greatly amplified by tau expression (Bloom, 2014; Nisbet et al., 2015). Removing endogenous tau prevents Aβ-associated cognitive impairments (Guerrero-Muñoz et al., 2015). Aβ-induced upregulation of intracellular calcium levels is a key upstream event for the formation of tauopathy and dislocation in the dendritic compartment (Bloom, 2014; Zempel and Mandelkow, 2015). Furthermore, pyroglutamylated Aβ, an important form of Aβ, induces tau-dependent toxicity and propagates in a prion-like manner (Nussbaum et al., 2012).

### Nuclear Paraspeckles Assembly Transcript 1 Induces Tau Dephosphorylation

NEAT1 is vital for nuclear paraspeckles, and it regulates nuclear bodies, chromatin remodeling, microtubules (MTs) stability and gene expression (Martin et al., 2013b). Recent studies have demonstrated that NEAT1 is correlated to neuronal loss and neurodegenerative disorders (Lo et al., 2016; Sunwoo et al., 2017). Knockdown of NEAT1 increases the expression of p-tau and dysfunction of MTs through Frizzled Class Receptor 3 (FZD3)/CSK3β/p-tau pathway (Kickstein et al., 2010). Interestingly, metformin increases NEAT1 expression, and leads to decreased FZD3 expression and dephosphorylation of tau (Zhong et al., 2017). Additionally, NEAT1 modulates Aβ via regulating miR-124/BACE1 axis (Zhao et al., 2020b).

### Linc00507 Induces Tau Hyperphosphorylation

Linc00507, first described in the Mammalian Gene Collection Program, is expressed in a cortex-specific manner in non-human primates and humans (Strausberg et al., 2002; Ransohoff et al., 2018). Linc00507 is upregulated in the hippocampus and cerebral cortex of APP/PS1 mice, which subsequently triggers the p25/p35/GSK3β activation and leads to tau-pathology. In addition, linc00507 functions as an endogenously competing RNA (ceRNA) that directly binds to miR-181c-5p, inducing the upregulation of MAPT and tau tubulin kinase 1 (TTBK1) (Strausberg et al., 2002; Mills et al., 2016).

## LncRNA and Loss of Synaptic Homeostasis

### Loss of Synaptic Homeostasis and AD

An analysis of post-mortem brain tissues from AD patients has revealed significant synapse loss (Henstridge et al., 2015; de Wilde et al., 2016). Restoring excitatory synaptic transmission in the hippocampus can effectively ameliorate the cognitive deficits in animal models with AD (Nisticò et al., 2012). The synaptic pathology correlates with clinical manifestations of AD and parallels the cognitive decline (Selkoe, 2002; Kashyap et al., 2019). In addition, dramatic synaptic loss is the first indicator of AD progression even in the earliest stages of AD. Increasing evidence reveals that synaptic dysfunction may be due to soluble Aβ, phosphorylated tau accumulation and mitochondrial free radicals at synapses (John and Reddy, 2021; Pereira et al., 2021). The physiological levels of Aβ may enhance neuronal activity by presynaptic potentiation and further facilitate Aβ production, and ultimately induces negative postsynaptic regulation of excitatory synaptic transmission (Palop and Mucke, 2010). However, excessive Aβ may lead to the dysfunction of pre-synapses consisting of axonal transport, synaptic vesicle cycling and neurotransmitter release. The interaction of Aβ oligomers and postsynaptic compartment of excitatory synapses with high affinity leads to synaptic plasticity impairment (Selkoe, 2002; Palop and Mucke, 2010; Chen et al., 2019). The abnormal accumulation and mislocalization of tau disrupts the microtubule-based cellular transport and impedes the trafficking of essential cargo, leading to decreased mitochondrion-dependent ATP production, calcium buffering and synapse loss (Forner et al., 2017; John and Reddy, 2021). In addition, ApoE and its receptor regulate synaptic functions at both pre- and postsynaptic sites, amongst which ApoE4 induces neuronal dysfunction at the earliest stages of AD (Lane-Donovan and Herz, 2017; Zhao et al., 2020a). Furthermore, the dysfunction of AMPA receptors (AMPAR) trafficking impairs neuronal circuit formation and causes long-term depression, which contributes to the symptoms of AD (Jurado, 2017; Ma et al., 2020).

### BC200 Impairs Synaptic Functions

BC200 is selectively expressed in neurons and delivered to the dendrites to regulate the synthesis of local proteins (Yan et al., 2020), and maintains the long-term plasticity (Muslimov et al., 1997). The mislocalization and overexpression of BC200 contributes to dendrites impairment in AD. The level of BC200 in affected brain areas closely correlates with the synaptic impairment and the severity of AD (Muddashetty et al., 2002; Bassell and Twiss, 2006). In addition, the somatodendritic distribution of BC200 is altered in severe AD (Muddashetty et al., 2002; Bassell and Twiss, 2006). Furthermore, BC200 binds to eukaryotic initiation factor 4A (eIF4A) and other RNA-binding proteins to regulate the levels of post-synaptic dendritic microdomains, including FMRP, synaptotagmin binding cytoplasmic RNA interacting protein (SYNCRIP) and poly (A)-binding protein (PABP) (Zalfa et al., 2005; Mus et al., 2007; Duning et al., 2008).

### BDNF-AS Damages Synaptic Plasticity

Brain-derived neurotrophic factor (BDNF) plays a crucial role in neuronal survival and synaptic plasticity and promotes the synapse growth, which consequently regulates learning and memory function (Lu et al., 2014; Petukhova et al., 2019). BDNF-AS is a conserved non-coding antisense RNA transcript, and modulates synaptic structure and functions via interacting with BDNF mRNA (Alsina et al., 2001). BDNF is decreased in most neurodegenerative disorders (Ji et al., 2010), however, some studies show increased BDNF in the post-mortem brain tissue with AD (Ventriglia et al., 2013). BDNF-AS forms an *in vivo* RNA-RNA duplex with BDNF mRNA and decreases the protein level of BDNF, while BDNF-AS inhibition upregulates the level of BDNF (Alsina et al., 2001). Moreover, BDNF-AS downregulates the level of BDNF mRNA through interfering chromatin at its locus (Alsina et al., 2001).

## LncRNA and Mitochondrial Dysfunction

### Mitochondrial Dysfunction and AD

Mitochondrial dysfunction is revealed as one of the earliest features of AD (Serý et al., 2013). The brain consumes nearly 20% of the total basal oxygen budget to support ATP demands, and it is susceptible to oxidative stress and energy shortage due to mitochondrial dysfunction (Galluzzi et al., 2012; Perez Ortiz and Swerdlow, 2019). Several studies suggest that bioenergetic deficits precede the accumulation of Aβ and tau, and are exacerbated with these aggregated proteins (Galluzzi et al., 2012; Tyumentsev et al., 2018). Moreover, it is found that restoration of the activity of phosphatase and tensin homolog (PTEN) induced putative kinase 1 (PINK1) improves the cognitive functions and lowers Aβ production in AD mice (Tyumentsev et al., 2018; Lim et al., 2020).

### Nuclear Enriched Abundant Transcript 1 Induces Mitochondrial Impairment

NEAT1 is a lncRNA transcribed from the multiple endocrine neoplasia type 1 (MEN1) gene, known as a scaffold for paraspeckles. NEAT1 plays a vital role in the formation and maintenance of paraspeckles (Cadonic et al., 2016). NEAT1 is upregulated during aging in the APP/PS1 transgenic mouse model and in the temporal cortex and hippocampus of AD mice (Liu et al., 2014; Huang et al., 2020). Knockdown of NEAT1 ameliorates cognitive impairments and improves hippocampal memory formation, and its overexpression exacerbates the progression of AD pathology and cognitive impairment in AD mice (Zhou et al., 2018b; Cao et al., 2019). The underlying mechanisms of NEAT1 in AD remain undefined. Recent studies show that NEAT1 interferes with mitochondria through PINK1 in AD models (Zhou et al., 2018b). NEAT1 promotes the degradation of PINK1 and impairs PINK1-dependent autophagy, leading to the dysfunction of autophagy signaling and inducing the amyloid accumulation and mitochondrial impairment (Zhou et al., 2018b; Lim et al., 2020). In addition, NEAT1 regulates Aβ accumulation in AD mice through interacting with miR-124 and miR-107, and knockdown of NEAT1 attenuates Aβ-induced neuronal damage (Zhou et al., 2018b; Butler et al., 2019; Ke et al., 2019).

## LncRNA and Neuronal Apoptosis

### Neuronal Apoptosis and AD

Neuronal apoptosis plays an important role in central nervous system, and the perturbation of apoptosis is involved in the neurodegenerative diseases including AD (Gu et al., 2018). Caspases act as both initiator and executor of apoptosis, and at least 7 caspases have been involved in AD including caspase-1, 2, 3, 6, 8, 9, and 12. For instance, the level of caspase-1 mRNA is upregulated in AD brain extracts (Qian et al., 2015). The deficiency of caspase-2 protects several neuronal subtypes from Aβ-induced apoptotic death *in vitro* (Desjardins and Ledoux, 1998), and caspase-3 is increased in AD brain and is activated in Aβ-treated neuronal cultures (Gervais et al., 1999). Previous reports have shown that many DNA fragmentation in post-mortem brains of AD patients, which indicates the activity of apoptosis in AD (Lassmann et al., 1995). All these data suggest that neuronal apoptosis dysregulation mediates the pathogenesis of AD.

### Early B Cell Factor 3 Antisense RNA Induces Neuronal Apoptosis

EBF3-AS, a 2-exon RNA transcribed from the opposite strand of the protein-coding gene Early B cell factor 3 (EBF3), is abundantly expressed in brain (Zhao et al., 2019). EBF3 is thought to be a target gene of EBF3-AS and is potentially associated with age in LOAD (Magistri et al., 2015). Previous studies have revealed that EBF3 homologs are essential for survival and dysfunction of EBF3 correlates to a range of nervous system developmental defects including perturbation of neuronal development and migration (Belbin et al., 2011). EBF3-AS and EBF3 are upregulated in the hippocampus of AD mice, and knockdown of EBF3-AS and EBF3 inhibits the apoptosis induced by Aβ (Chao et al., 2017). These results suggest that EBF3-AS induces neuronal apoptosis in AD, supporting EBF3-AS as a new target for AD treatment.

### Natural Antisense Transcript Against Rad18 Promotes Neuronal Apoptosis

NAT-Rad18, with a length of 509 nucleotides, plays a crucial role in DNA repair, and is directly responsible for the specific mono-ubiquitylation of the polymerase adapter PCNA (Lloyd et al., 2006; Parenti et al., 2007). NAT-Rad18 is universally expressed in the brain, especially in the cerebellum, brainstem, spinal cord, olfactory bulb, cortex, hippocampus and striatum (Flores et al., 2018). The upregulation of NAT-Rad18 renders cells more sensitive to a wide spectrum of DNA-damaging agents (Harvey et al., 2004), which may be part of a complex transcriptional and post-transcriptional genomic program underlying Aβ-neurotoxicity.

### Metastasis-Associated Lung Adenocarcinoma Transcript 1 Reduces Neuronal Apoptosis

MALAT1 is a long intergenic non-coding RNA that is located on chromosome 11q13 and consists of 8,828 nucleotides (Tateishi et al., 2000). Emerging evidence suggests a neuroprotective function of MALAT1 *via* inhibiting neuroinflammation. MALAT1 is decreased in Aβ1−42 treated neurons, and induces the neurite outgrowth (Ji et al., 2003; Ma et al., 2019). Overexpression of MALAT1 reduces neuronal apoptosis and alleviates neuronal injury (Zhuang et al., 2020), and knockdown of MALAT1 promotes neuronal apoptosis and represses neurite growth (Ji et al., 2003). Additionally, MALAT1 modulates miR-125b expression and consequently suppresses neuronal apoptosis and inflammation (Ji et al., 2003; Ma et al., 2019).

### Taurine Upregulated Gene 1 Facilitates Neuronal Apoptosis

TUG1 is a novel lncRNA with 6.7-kb nucleotides located on the chromosome 22q12, and is involved in neuronal apoptosis, proliferation, cell cycle and metastasis (Li et al., 2020a). Recent studies have revealed the important role of TUG1 in AD through controlling the neuronal apoptosis. TUG1 silencing decreases cellular apoptosis in Aβ_25−35_-treated hippocampal neurons, and consequently improves spatial learning and memory of AD mice (Guo et al., 2020). In addition, TUG1 acts as miR-15a sponge and regulates neuronal apoptosis via the proteolytic cleavage of crucial proteins (Guo et al., 2020; Li et al., 2020b).

### Wilms Tumor 1 Homolog Antisense RNA Inhibits Neuronal Apoptosis

WT1-AS, a lncRNA located on chromosome 11p13, is important in regulating transcription, apoptosis and RNA metabolism (Zhang et al., 2019a; Wu et al., 2021). WT1-AS is downregulated in Aβ_25−35_ treated SH-SY5Y cells, and overexpression of WT1-AS inhibits WT1 expression and reverses the deleterious effects of Aβ_25−35_ (Toska and Roberts, 2014). In addition, WT1-AS inhibits apoptosis via reducing WT1 expression or suppressing miR-375 expression (Toska and Roberts, 2014).

## LncRNA and Neuroinflammation

### Neuroinflammation and AD

Neuroinflammation is a response to various stimuli and consists of glia cells, lymphocytes, monocytes and macrophages, which directly contributes to the pathogenesis and progression of AD (Maccioni et al., 2020). Neuroinflammation acts as a “double-edged sword” in the central nerve system (Cortés et al., 2018; Maccioni et al., 2020). The balance between neuronal damage and inflammation is mainly regulated by glia cells (Maccioni et al., 2020). Microglia functions as resident phagocytes to dynamically monitor the environment, and contributes to the brain development and synaptic pruning (Frost and Schafer, 2016; Colonna and Butovsky, 2017). Astrocytes are shown to maintain brain homeostasis, protect neural circuits and repair injuries (Sofroniew and Vinters, 2010; Cai et al., 2017b). Dysfunction of astrocytes induces tau hyperphosphorylation and NFT formation and failure of Aβ clearance (Yan et al., 2013; Leyns and Holtzman, 2017). Moreover, astrocytes are the most important energy regulators in CSF, and astrocyte metabolic dysfunction is considered as an initiating factor in AD (Yan et al., 2013).

### Maternally Expressed Gene 3 Reduces Neuroinflammatory Injury

MEG3 locates on chromosome 14 in humans and acts as a mediator in inflammation. MEG3 plays a key role in various biological processes including microglia activation and inflammatory response (Kobayashi et al., 2000; Meng et al., 2021). Upregulation of MEG3 inactivates astrocyte through inhibiting the PI3/Akt pathway, and improves the spatial memory in AD rats (Yi et al., 2019). MEG3 is also a direct target of miR-7a-5p, and overexpression of MEG3 reduces miR-7a-5p and promotes microglia activation (Meng et al., 2021).

### MALAT1 Attenuates Neuroinflammation

Accumulating evidence indicates the neuroprotective and anti-inflammatory role of MALAT1 in neurodegenerative diseases (Zhou et al., 2018a; Masoumi et al., 2019). MALAT1 inhibits the inflammation-associated miRNAs levels, and attenuates neuroinflammation in AD (Ma et al., 2019). MALAT1 is also decreased in Aβ_1−42_ treated cells and inhibits neuronal apoptosis (Ma et al., 2019).

### Other LncRNA With AD

Glial cell line-derived neurotrophic factor (GDNF) is a neurotrophic peptide, and is known as a neurotropin to promote the survival and differentiation of midbrain dopaminergic neurons (Ledda et al., 2007; Airavaara et al., 2011). Glial cell line-derived neurotrophic factor opposite strand (GDNFOS) is a cis-natural antisense transcribed from the opposite strand of GDNF gene (Cortini et al., 2019). In patients with AD, the level of mature GDNF is increased in CSF and decreased in serum, while GDNFOS1 is upregulated in cerebellum (Straten et al., 2009; Airavaara et al., 2011). MAGI2-AS3 is significantly increased in Aβ_25−35_ induced neuronal cells and in AD patients, and knockdown of MAGI2-AS3 attenuates neurotoxicity and neuroinflammation (Wang et al., 2020). LncRNA X-inactive specific transcript (XIST) is a functional lncRNA which plays an important role in the development and progression of many malignant tumors (Yi et al., 2019). The expression of XIST is significantly increased in AD models and silencing XIST negatively regulates the expression of miR-124 and promotes BACE1 expression (Du et al., 2017). Ribonuclease P RNA component H1 (RPPH1) is an RNA component of the RNase P ribonucleoprotein, which cleaves tRNA precursor molecules to generate the mature tRNA (Yue et al., 2020). Overexpression of RPPH1 increases the density of dendritic spine in hippocampal neuron (Cai et al., 2017a), which suggests a protective role of RPPH1 in the early stage of AD. Small nucleolar RNA host gene 1 (SNHG1) is upregulated in Aβ_25−35_ treated cells and knockdown of SNHG1 attenuates Aβ_25−35_ induced mitochondrial dysfunction and cell apoptosis (Cai et al., 2017a; Wang et al., 2019a). Recent studies have shown that knockdown of the lncRNA antisense non-coding RNA in the INK4 locus (lnc-ANRIL) inhibits apoptosis and promotes neurite outgrowth in a cellular model of AD (Zhou et al., 2020).

### LncRNA in Clinical AD Management and Perspective

LncRNAs are relatively stable, which indicates that the serum or CSF lncRNAs might be promising biomarkers and therapeutic targets for AD diagnosis and treatment (Table 1). The concentration of BACE1 in CSF and plasma shows a good diagnostic value in AD patients (Shen et al., 2018; Lopez-Font et al., 2019). Therapeutic strategies targeting BACE1 have been extensively developed but discontinued due to futility or safety reasons (Ghosh and Osswald, 2014; Hampel et al., 2021). BACE1-AS becomes an attractive biomarker for AD, and the level of BACE1-AS is upregulated in the brain and plasma of AD patients (Faghihi et al., 2008; Feng et al., 2018) but significantly decreased in pre-AD cases (Fotuhi et al., 2019). Overexpression of NDM29 is observed in AD postmortem cerebral cortex samples (Massone et al., 2012). 51A is overexpressed in AD post-mortem samples and shows an active role in altering SORL1 expression in AD patients and a positive correlation with Aβ production compared with that in healthy controls (Ciarlo et al., 2013). 17A is upregulated in cerebral cortices in AD patients and is specifically overexpressed in AD patients rather than other neurodegenerative diseases (Massone et al., 2011). The level of BC200 in cortical areas is increased in brains from AD patients, and is reduced in normal aging individuals (Mus et al., 2007). However, it is also shown that the plasma levels of 17A, 51A and, BC200 are not significantly affected in AD patients compared with those in age-matched controls (Feng et al., 2018). These inconsistent results may be attributed to relative smaller sample size and different disease stages. Larger-scale trials are needed to elucidate the lncRNA profile in AD.

**Table 1:** Potential lncRNA biomarkers in AD patients.

**Related lncRNA**	**Regions of AD patients**	**Biological function**	**References**
BACE1-AS↑/↓	Brain, plasma	Upregulating BACE1 mRNA stability; Altering Aβ aggregation pattern increasing Aβ expression.	Faghihi et al., 2008; Fotuhi et al., 2019
NDM29↑	Cerebral cortex	Promoting the cleavage activity of BACE andγ-secretase; Increasing Aβ secretion and Aβ_x−42_/Aβ_x−40_ ratio.	Massone et al., 2012
51A↑	Cerebral cortex and plasma	Downregulating SORL1; Increasing production and accumulation of Aβ.	Massone et al., 2011; Ciarlo et al., 2013
17A↑	Cerebral cortex	Impairing the GABAB signaling pathway	Massone et al., 2011
BC200↑	Cerebral cortex	Inducing Aβ production and amyloid deposition; Maintaining the long-term synapse plasticity	Mus et al., 2007

*The arrows next to lncRNA indicates up/down-regulation in AD patients*.

## Conclusion

Up to now, numerous lncRNAs have been identified to be associated with AD, but it is only a tip of the iceberg. LncRNAs play a critical role in the AD pathogenesis including amyloid production, Tau hyperphosphorylation, mitochondrial dysfunction, synaptic impairment and neuroinflammation. However, how lncRNAs function at molecular and cellular levels remains a huge challenge, and the biological characteristics and underlying mechanisms of lncRNAs in AD still need to be elucidated. Undoubtedly, further investigation of lncRNAs lights a new beacon for clinical diagnosis and treatment of AD.

## Data Availability Statement

The original contributions presented in the study are included in the article/supplementary material, and further inquiries can be directed to the corresponding author.

## Author Contributions

ZL, YC, JJ, YX, and XZ wrote the paper. All authors read and approved the final manuscript.

## Funding

This research was supported by the National Nature Science Foundation of China (81971009, 81630028, and 81920108017), the Fundamental Research Funds for the Central Universities (021414380519), the Natural Science Foundation of Jiangsu Province (SBK2021021574), and the National Key Research and Development Program of China (2018YFC1704400).

## Conflict of Interest

The authors declare that the research was conducted in the absence of any commercial or financial relationships that could be construed as a potential conflict of interest.

## Publisher's Note

All claims expressed in this article are solely those of the authors and do not necessarily represent those of their affiliated organizations, or those of the publisher, the editors and the reviewers. Any product that may be evaluated in this article, or claim that may be made by its manufacturer, is not guaranteed or endorsed by the publisher.

## Abbreviations

AD: Alzheimer's disease
ncRNAs: non-coding RNAs
lncRNAs: long non-coding RNAs
LOAD: late onset of AD
EOAD: the early onset of AD
APP: amyloid precursor protein
Aβ: beta amyloid
APOE4: apolipoprotein genotype E4
TREM2: triggering receptor expressed on myeloid cells 2 gene
BACE1-AS: β-site APP cleaving enzyme-1 antisense
sAPPα: secreted amyloid precursor protein α
AICD: APP intracellular domain
FDA: Food and Drug Administration
CSF: Cerebrospinal Fluid
BDNF-AS: brain-derived neurotrophic factor antisense
UTRs: untranslated regions
SORL1: Sortilin-related receptor 1
TGN: trans-Golgi network
GPR51: G-protein-coupled receptor 51 gene
BC200: brain cytoplasmic 200
FMRP: fragile X syndrome protein
NDM29: neuroblastoma differentiation marker 29
MAP: microtubule-associated proteins
MAPT: microtubule-associated protein tau
PHF: paired helical filaments
NFTs: neurofibrillary tangle
NEAT1: nuclear paraspeckles assembly transcript 1
MTs: microtubules
FZD3: Frizzled Class Receptor 3
ceRNA: competing endogenous RNA
MCI: Mild Cognitive Impairment
eIF4A: eukaryotic initiation factor 4A
SYNCRIP: synaptotagmin binding cytoplasmic RNA interacting protein
PABP: poly (A)-binding protein
MEN1: multiple endocrine neoplasia type 1
EBF3-AS: Early B cell factor 3 antisense RNA
NAT-Rad18: natural antisense transcript against Rad18
MALAT1: metastasis-associated lung adenocarcinoma transcript 1
TUG1: taurine upregulated gene 1
WY1-AS: Wilms' tumor 1
GDNFOS: glial cell line-derived neurotrophic factor opposite strand
XIST: X-inactive specific transcript
SNHG1: small nucleolar RNA host gene 1
RPPH1: Ribonuclease P RNA component H1
lnc-ANRIL: lncRNA antisense non-coding RNA in the INK4 locus.
